# Chikungunya Virus: Pathophysiology, Mechanism, and Modeling

**DOI:** 10.3390/v9120368

**Published:** 2017-12-01

**Authors:** Vaishnavi K. Ganesan, Bin Duan, St Patrick Reid

**Affiliations:** 1 Mary & Dick Holland Regenerative Medicine Program, University of Nebraska Medical Center, Omaha, NE 68198, USA; vaishnavi.ganesan@unmc.edu; 2 Division of Cardiology, Department of Internal Medicine, University of Nebraska Medical Center, Omaha, NE 68198, USA; 3 Department of Surgery, College of Medicine, University of Nebraska Medical Center, Omaha, NE 68198, USA; 4 Department of Pathology and Microbiology, College of Medicine, University of Nebraska Medical Center, Omaha, NE 68198, USA

**Keywords:** arboviruses, pathogenesis, vaccine, 3D organoid

## Abstract

Chikungunya virus (CHIKV), a mosquito-transmitted alphavirus, is recurring in epidemic waves. In the past decade and a half, the disease has resurged in several countries around the globe, with outbreaks becoming increasingly severe. Though CHIKV was first isolated in 1952, there remain significant gaps in knowledge of CHIKV biology, pathogenesis, transmission, and mechanism. Diagnosis is largely simplified and based on symptoms, while treatment is supportive rather than curative. Here we present an overview of the disease, the challenges that lie ahead for future research, and what directions current studies are headed towards, with emphasis on improvement of current animal models and potential use of 3D models.

## 1. Introduction

### 1.1. Chikungunya Fever and CHIKV

Chikungunya, a mosquito-borne disease endemic to tropical regions, has emerged as an epidemic threat over the past 15 years. It infects over one million people per year and causes debilitating joint pain [1]. The name “chikungunya” derives from a Makonde phrase meaning “that which bends up” or “to become contorted”, referring to the bent posture of affected patients. Chikungunya fever (CHIKF) is caused by Chikungunya virus (CHIKV), a pathogen of the genus alphavirus and the family *Togaviridae* [2]. Within this genus, there are 30 species of arthropod-carried alphaviruses (also referred to as arboviruses, stemming from *ar*thropod-*bo*rne viruses), all sharing seven specific antigenic complexes [3]. CHIVK is closely related to several other alphaviruses, including Ross River virus, Barmah Forest virus, o’nyong-nyong virus, the Sindbis group of viruses, and the Mayaro virus, all of which are known to cause arthritis [4]. CHIKV has three genotypes: Asian, West African, and East Central South African, all named after their geographical distributions. The virus itself is a positive-sense, single-stranded RNA virus approximately 11.8 kb long [5]. It has an icosahedral capsid which is covered by a lipid layer, a diameter of approximately 65 nm, and is sensitive to temperatures greater than 58 °C [6]. It contains two open reading frames (ORFs), one on the 5′ end and the other on the 3′ end [7], with the ORF at the 5′ end producing four non-structural proteins (nsP 1–4) and the ORF on the 3′ end producing the structural proteins, which are composed of a capsid protein, two envelope glycoproteins (E1 and E2), and 2 small cleavage products (E3 and 6K) [8]. 

### 1.2. History/Origins

CHIKV was not recognized in the early 19th century, but what we now know as CHIKF was. It was not until the early 1950s that CHIKV was first characterized in East Africa [9]. It was more specifically outlined in southern Tanzania in 1952, where it was first isolated in a human [10]. Following its discovery, the disease was largely confined to pockets of land in Asia and Africa. The disease was marked by long gaps of inactivity interspersed with sudden outbreaks [11], though this characterization has been disputed [12].

It was not until the late 1990s and early 2000s that CHIKV began to re-emerge on a global scale. Many of these recent epidemics differed from those previously reported in both their increased scale and more rapid movement, with many originating from migrant populations moving from areas with endemic CHIKV. Malaysia had an outbreak in 1998 that was present primarily in adults and speculated to be re-introduced through the movement of workers [13], while Indonesia had a major outbreak in 2001–2003 after a 20-year hiatus of epidemic CHIKV [14]. The 2005 Indian epidemic, one of the most severe of the recent outbreaks, provided a possible template for envisioning future outbreaks and their consequences. The outbreak occurred after a 32-year gap of epidemic CHIKV in the region and eventually affected 1.3 million people [15]. The national burden from the outbreak was estimated to be nearly 26,000 disability-adjusted life years (DALYs) lost, equating to approximately 45.26 DALYs per million people [16]. 2005 also saw a severe CHIKV outbreak on Réunion, an island found in the Indian Ocean east of Madagascar. This outbreak affected one-third of the island’s population [17] and was notable in that its neural, hepatic, and myocardial symptoms led to an unusually high mortality rate as compared to previous CHIKV outbreaks [18]. Table 1 summarizes recent large-scale outbreaks of CHIKV in the 2000s.

As global travel has increased, there has been an increase of CHIKF cases described in Western nations. In September 2007, a small outbreak of CHIKV stemming from an imported case was noted in Italy and was especially concerning as it was spread via local infected mosquitoes [19]. There were no locally acquired cases in the USA from 1995 to 2009, but there were reports of imported cases during that time [20]. Similarly, imported cases coming from the Caribbean were noted in Spain in 2013 [21]. Of note, various autochthonous outbreaks have occurred in Europe, highlighting the ability of the virus to cause disease in these more temperate climates [22,23]. CHIKV was not noted in the Americas before 2013, but has since rapidly spread throughout the region. In the years following the emergence of CHIKV in the Americas, it has caused between 795,000 and 1.1 million cases, and spread to 43 countries and territories. During a 2014 outbreak on the islands of Martinique and Guadeloupe, an estimated 308,000 people were affected [24], and from October 2014 to March 2015, 66,000 people in French Polynesia were infected, equating to an infection rate of 25% [24]. In addition, CHIKV establishment in the Americas was followed by a sharp increase in travel-related CHIKF with upwards of 1600 passengers coming to the US with CHIKF in the year following its emergence, compared to a previous average of 28.

### 1.3. Symptoms

Though it can appear similar to dengue fever and Zika, CHIKF is indicated by several characteristic symptoms. CHIKV is remarkable in that it creates symptoms in a higher proportion of infected individuals as compared to other alphaviruses, with 10–70% of persons living in an affected area becoming infected, and 50–97% of the infected developing a clinical presentation [1]. Symptoms typically appear after an incubation time of 4–7 days [25]. The disease has a more severe effect on neonates and the elderly [26], and in neonates it is associated with encephalitis [27]. The mortality rate is five times higher in individuals 65 and above compared to those less than 45 years of age [28]. The disease course is divided into an acute stage, lasting approximately one week, and a chronic stage, also known as the persistent stage, which can last from months to years. Acute fever and polyarthralgia are highly indicative of an infection [25], with arthralgia appearing in 30–90% of cases [5,6]. This joint pain is often bilateral, symmetric, and debilitating. There are occasional ophthalmic [29], neurological [30], and cardiac [31] symptoms.

Chronic CHIKV infection is less studied but represents a significant health complication for those afflicted and a public health problem for their communities. In a case report investigating the Réunion outbreak, it was reported that two years post-infection, 43–75% of patients continued to have symptoms attributed to their infection [32]. The most prominent symptoms one month post-infection were rheumatism (75%) and fatigue (30%), with joint pain, fatigue, and neuritis being present after ten months [33]. Another study suggests that 50% of people infected with CHIKV will go on to experience chronic pain [34]. Chronic CHIKF demonstrates specificity to bone and joint tissue with symptoms such as rheumatoid arthritis and ankylosing spondylitis [35].

In part due to the symptoms noted, CHIKV is associated with low mortality but high morbidity. During the Réunion outbreak, a case-fatality rate of 1/1000 was reported [36]. However, during the outbreaks occurring in 2004–2008, an increase in death rates was noted, with the deaths following the previously established pattern of affecting the elderly proportionately more than younger adults [37]. It has been argued that the fatality of CHIKF has been underestimated [38].

When laboratory tests are performed, the primary lab finding is lymphopenia, delineated as having <1000 lymphocytes/mL^3^ [6]. Along with the lymphopenia, there is occasional leukopenia, elevated liver enzymes, anemia, elevated creatinine, elevated creatinine kinase, and hypocalcemia [6]. The acute stage of CHIKV is noted to have a high viremic load, with an average of 10^7^ pfu/mL [6].

## 2. Pathophysiology

### 2.1. Transmission

CHIKV is transmitted in two different cycles: urban and sylvatic. The urban cycle refers to transmission from human to mosquito to human, while sylvatic transmission is animal to mosquito to human [17]. The sylvatic cycle is the primary form of maintenance in Africa [39]. CHIKV elsewhere in more densely populated areas is primarily maintained in an urban cycle, in which humans act as the major hosts and mosquitos of the genus *Aedes* act as vectors [17]. Specifically, the virus is primarily transmitted by *Aedes aegypti* and *Aedes albopictus.* The principal vector in CHIKV transmission has historically been *Ae. aegypti*, but *Ae. albopictus* acted as the major vector in several recent outbreaks in Réunion, Europe, and Gabon [17], although *Ae. Aegypti* continues to be an important viral vector as seen during the Caribbean outbreak in 2013 [40]. The adaptation to *Ae. albopictus* has been theorized to be due in part to a lack of sufficient *Ae. aegypti* vectors [41], with a mutation in the E1 envelope protein allowing *Ae. albopictus* to serve as a competent vector. The A226V mutation in the E1 envelope protein increased fitness of CHIKV in *Ae. albopictus* and improved transmissibility to vertebrate species [17]. Mother-to-child vertical transmission has been postulated for the post-2005 incidences [26], being especially deleterious when the mother is infected up to four days postpartum [42], though this hypothesis has been disputed [43].

### 2.2. Route of Infection

CHIKV has an established route of infection in humans, with certain cell types being particularly susceptible to infection. Those cells prone to infection and viral production include human epithelial and endothelial cells, primary fibroblasts, and monocyte-derived macrophages, while lymphoid and monocytoid cells, primary lymphocytes and monocytes, and monocyte-derived dendritic cells did not demonstrate CHIKV replication [44]. Although this claim can be disputed, since CHIKV-positive monocytes have been detected in vivo from CHIKV-infected patients [45]. After the first round of replication there is a host immune response, but the virus goes to the lymph nodes and then to other tissues via the circulatory system [46]. Replication at other tissues leads to the viremic phase of the disease, which is additionally when mosquitoes can transmit the disease via blood.

### 2.3. Innate Immune Response

The immune response to CHIKV infection has been partially elucidated, but significant portions remain unknown. Upon infection, CHIKV indirectly stimulates production of type I interferon (IFN) via activation of non-hematopoietic cells, including primarily fibroblasts, an action which is essential for clearing CHIKV from the body [47]. While interferon regulatory factors (IRFs) 3 and 7 acted in a redundant manner in adults, neonates lacking in either factor were significantly more prone to infection [47]. CHIKV also appears to induce a signaling cascade by activating interferon promoter stimulator 1 (IPS-1), leading to the buildup of IRF3-dependent mRNAs while also blocking these mRNAs from encoding proteins; the mechanism for this impediment is as-yet unknown [48].

CD8+ lymphocytes are found in skin rashes of acute patients, while CD4+ T-cells comprise the majority in synovial effusions of chronic patients [49]. This evidence was supported by experiments done by Wauguier et al., which found that there was an activation of CD8+ T-cells early in the infection, followed by CHIKV-induced apoptosis of CD4+ T-cells, with later parts of the acute stage demonstrating a higher CD4+ T-cell count [50]. BST-2 (bone marrow stromal antigen 2) may protect lymph tissues and regulate CHIKV-induced host inflammatory responses [51].

There is evidence that inflammation stemming from CHIKV infection has consequences for osteoblast and osteoclast proliferation and function, which may contribute to the effects of chronic CHIKV. Several of the cytokines associated with infection, such as TNF-α, IL-6, and IL-1, also promote osteoclast activity and have been associated with osteoclastogenesis [52].

The brain is noted for having its own innate immune response. As noted previously, though neurologic symptoms are uncommon, they can be severe, particularly in neonates. The brain has several methods for combating CHIKV infection. Mouse models demonstrated that astrocytes and oligodendrocytes were particularly susceptible to infection, while microglial cells were more resistant [53]. In astrocytes, CHIKV induced apoptosis via the external caspase-9 pathway. In response to CHIKV, microglial cells produce cytokines and pro-apoptotic molecules including IL-12, IFN-α, and TNF-α [54].

## 3. Diagnosis/Treatment

### 3.1. Diagnosis

Diagnosis can be delayed due to the possible confusion of symptoms with those of dengue fever or Zika. Fever and polyarthralgia give 84% sensitivity, 71% positive predictive value (PPV), and 83% negative predictive value (NPV) [6]. Enzyme-linked immunosorbent assays (ELISA) can be used to confirm the presence of anti-CHIKV antibodies, with IgM antibody levels highest three to five weeks post-infection and persisting for up to two months. PCR can also be used to genotype the virus.

### 3.2. Treatment

Current treatment focuses on lessening the severity of symptoms rather than curing the disease. Treatment primarily involves the use of antipyretics and NSAIDs [5]. However, there have been no studies to systematically evaluate the efficacy of these treatments [6], and the symptoms may dissipate without intervention. The use of corticosteroids for acute stage treatment has met mixed success and is hesitantly used due to the possibility of worsening symptoms post-treatment [55]. Maintaining proper fluid levels is of particular importance. There is also emerging evidence that drugs that impede cholesterol transport, such as the class II cationic amphiphilic compounds U18666A and imipramine, may be effective against CHIKV membrane fusion, and has potential for working against other arboviruses [56].

For severe chronic arthralgia, disease-modifying anti-rheumatic drugs (DMARDs), including methotrexate, hydroxychloroquine, or sulphasalazine, have been proposed [57]. Similar to the acute treatments, the systematic efficacy of DMARDs for chronic treatment is unknown [58], though there have been reports describing positive outcomes with cessation of symptoms within 4–6 months [59].

## 4. CHIKV Infection and Models

Accurate modeling of the pathogenesis of CHIKV infection is critical for continued progress against the disease. Most in vitro and in vivo models of CHIKV have investigated the mechanisms of viral entry and replication, and the efficacy of various antiviral treatments. As CHIKV has been modeled with several different methods, there are several categorizations of CHIKV modeling. There is in vitro cell culture modeling, and mouse and non-human primate in vivo modeling. Within these various models, acute stage and chronic stage infection models also have been developed. Each model demonstrates unique facets of CHIKV pathogenesis while having unique shortcomings.

### 4.1. In Vitro Culture System

The most basic form of CHIKV modeling is in vitro cultures. Strictly speaking, this is not a model, but a culture system. In vitro culture systems form the basis of CHIKV analysis, but as such have significant drawbacks regarding clinical applications. They are the cheapest, fastest, and most accessible form of CHIKV analysis, making an ideal and commonly-used first step when testing antiviral therapies. A wide variety of cells are used for in vitro CHIKV cultures in order to assess the full scope of the disease, including primary human skeletal muscle myoblasts [60], human blood monocytes [45], and African Green monkey kidney (Vero-E6) cells [61]. Of particular interest is the use of primary human fibroblast-like synoviocytes [62] and human osteoblasts [63], given the known tropism of chronic CHIKV for bone and synovial tissues. However, while in vitro modeling has demonstrated some progress in accurately modeling the course of the disease, it still may not precisely predict in vivo behavior. For example, molecules known to have non-specific antiviral action (e.g., chloroquine and arbidol) showed some efficacy in vitro, but have not demonstrated clinical efficacy [5]. Similarly, in vitro testing demonstrated that some extracts from tropical plants, such as trigocherrings and trigowiin, have a good selectivity index against CHIKV, but in vivo models have yet to delineate their toxicity, tumorigenicity, and cellular mechanism [64]. Mefenamic acid, a component of non-steroidal anti-inflammatory drugs (NSAIDs), along with Ribavirin, demonstrated a significant decrease in the hypertrophic effects of CHIKV in mice livers and spleens, both in vitro and in vivo. It did so by impairing viral replication and entry [65].

### 4.2. Rodent Models

Mouse modeling is one of the most commonly used forms of animal modeling for CHIKV. Mice are relatively cheap, easily maintained, match well with many different mouse-specific reagents, are available with an abundance of different genetics lines, and are available in genetically-modified forms [66]. Mice have also been used to study transmission of CHIKV from mosquitoes [67]. As so many different types of murine model exist, mice models can be subdivided into several categories. Most broadly, murine models investigate either acute or chronic infection.

As delineated by Haese et al., acute models come in several subcategories, including neonatal, immunocompromised, and arthritic models. Neonatal mouse models are useful as they can provide more accurate models for the susceptibility of neonates to CHIKV infection. Neonatal mouse models have demonstrated that fibroblasts are the primary target for CHIKV [68], that inefficient type-1 IFN signaling is a risk factor for severe complications from infection [68], and that interferon-stimulating gene 15 (ISG-15) plays a critical, but not direct, role in CHIKV mitigation [69].

Research into the progress of chronic CHIKV infection also utilizes murine models, though this stage remains understudied. Mouse models have been used to show the presence of monoclonal antibodies (mAbs) specific to the capsid structural protein in chronic CHIKV. T- and B-lymphocyte-deficient recombination activating gene 1 (RAG1 −/−) knockout mice are often used for studying chronic CHIKV. This mouse model was used to demonstrate that CHIKV “establishes persistent infections in joint-associated tissues, that persistence of CHIKV RNA is associated with ongoing synovitis, and that the sites of CHIKV persistence and tissue burdens of CHIKV are controlled by adaptive immunity”, and that prophylactic and therapeutic treatments of mAbs have differing efficacies [70].

However, though they are very useful in many ways, CHIKV mouse models do have significant limitations. As yet, they have not been able to accurately simulate mother-child vertical transmission, determine how and why CHIKV more severely impacts the elderly, and determine the long-lasting consequences of chronic CHIKV [66]. Simulation of chronic CHIKV is particularly difficult, as mouse models expire quickly and may not demonstrate the full longevity and effects of persistent CHIKV.

Other rodent models aside from murine models are also being developed to study CHIKV. Baby hamster kidney cells (BHK-21-Clone 13) have been used since the 1970s to culture CHIKV [71]. Golden hamsters (*Mesocricetus auratus*) have demonstrated promise as future models for CHIKV, as they are highly susceptible to CHIKV infection and develop inflammations similar to humans, such as myositis and tenosynovitis [72].

### 4.3. Non-Human Primate Models

Along with mouse models, non-human primates (NHPs) provide the most commonly used animal models for CHIKV research. They provide key benefits as compared to murine models: more closely matching human physiology and demonstrating similar clinical symptoms. Macaques are among the most commonly used NHPs. They have been used to study over 70 infectious diseases, including HIV, poliomyelitis, malaria, and CHIKV [73]. Rhesus macaques (*Macaca mulatta*) have been in use for CHIKV research since the mid-1950s [74], and bonnet macaques (*Macaca radiata*) since at least the late 1960s [75], with cynomolgus macaques (*Macaca fascicularis*) also being utilized in the 21st century [76].

Broadly speaking, studies utilizing NHPs can be divided into two categories: those that study the mechanism of the disease and those that seek to test therapies. Immunocompetent cynomolgus macaques have demonstrated that NHPs can be utilized to model the symptoms of chronic CHIKV infection, including consequences for the musculoskeletal, lymph, and hepatic systems [77]. Cynomolgus macaques were also shown to be competent models for chronic CHIKV as the virus was harvested a month and a half post-infection from several key organs (muscle, liver, spleen). NHPs have also been used to glean more information about the effect of CHIKV on vulnerable populations. Pregnant rhesus macaques have demonstrated an absence of viral RNA in fetuses post-mortem [78]. Elderly macaques demonstrated a continuation of CHIKV in the spleen that was not present in younger mature macaques [79]. This continuation, along with a weaker immune system, may explain the higher morbidity found among older populations that is common during CHIKV outbreaks, though further studies are required.

Macaque models are being further developed to act as enzootic reservoirs for future therapies. They have been used to test the possibility of inactivated vaccines since the 1970s [80] and attenuated vaccines since the 1990s [81]. Macaques have been used to characterize the molecular mechanisms of anti-CHIKV antibodies, demonstrating that they target the E2 glycoprotein, capsid, and nsP1, nsP2, and nsP4 proteins [82]. Treatment of rhesus macaques with two monoclonal antibodies, CHK-152 and CHK-166, demonstrated a decrease in peripheral tissue infection [83]. When tested on rhesus macaques, a DNA vaccine expressing structural proteins E1–3 produced similar immunogenic effects as seen in human post-CHIKV infection [84].

Despite the demonstrated possibility of NHPs in modeling CHIKV, their use has certain difficulties. Consequently, due to the fact that NHPs are more difficult to utilize as compared to standard murine models, their use is not as widespread. NHPs are more expensive, require special restraints such as tethers and sedatives, and, along with other animal models, do not fully recreate the human microenvironment.

## 5. Challenges

One of the challenges preventing advancements in CHIKV research is the continuing lack of adequate models for testing, both human and animal. Though current murine and nonhuman primate models have established their utility, they still lack in certain areas. There remains a need to identify good models with the appropriate microenvironment to mimic the setting of an infection.

Up to the current day there is no animal model that completely shows the chronic rheumatoid syndrome for CHIKV, with current mice models demonstrating a destruction of tissue following infection [85]. As chronic CHIKV is a lifelong condition, models must be able to simulate the longevity of the disease. Further difficulties remain in inducing the severe joint damage caused by CHIKV in animal models. This lack of models to comprehensively simulate CHIKV infection may prevent forward progress to combating the disease. Treatments have not been completely tested due to a lack of relevant animal models. In particular, though there have been recent advancements, there is a dearth of animal models for testing the effects on bone and cartilage.

In addition, much of the work that remains in preventing future outbreaks of epidemic CHIKV centers on vector control of *Ae. aegypti* and *Ae. albopictus.* Traditionally, insecticides and larvicides have been used. However, mosquitoes have demonstrated an increasing resistance to insecticides [86]. There is evidence that some species of *Ae. albopictus* are developing resistance in the US to traditional insecticides such as DDT [87]. *Ae. albopictus* resistance against a variety of insecticides, including deltamethrin, beta-cypermethrin, cypermethrin, permethrin, dichlorvos, temephos, propoxur, and DDT, was reported throughout Guangzhou, China, with mortality rates ranging from 12 to 95% [88].

Underscoring all of these challenges is the expense of CHIKV research, prevention, and treatment. Due in part to the chronic nature of the disease, CHIKV is estimated to cost tens of millions of dollars in affected countries, with one estimate placing a burden of nearly $74 million for a single outbreak in Colombia in 2014 [89]. Vaccines in particular may have difficulties as they require a large amount of funding which may not be commercially viable.

## 6. Future Directions

One area in need of elucidation is the precise cellular mechanisms of the disease. While the coat glycoproteins required for viral entry are known structurally, less is known about possible target cell receptors and the precise mechanism for cell entry [86]. In addition, relatively more is known about innate immune responses to infection [51], which are largely in response to the acute state of the disease, but less is known about the nature of the associated chronic problems. It has been proposed that the changes to the immune response during the acute stage of CHIKV infection may facilitate the development of chronic problems [55], a theory which will require further studies with models to fully establish.

While animal models have been steadily improving in their ability to map out the behavior of CHIKV over the last few years, another area that potentially shows future promise is the use of 3D scaffolds and models. 3D models have recently shown use for studying infectious diseases. Thus far, their scope has been largely focused on gastrointestinal diseases, but the field is expanding [90]. Current research has begun developing systems for 3D tissue modeling of the brain, GI tract, kidney, liver, lung, and skin [91]. 3D models are advantageous for studying infectious disease as they accurately model human structures and can be composed of multiple cell types found in a particular organ. Organoids, 3D organ-like structures, have demonstrated use in modeling in vivo human pathology.

As the cell types susceptible to CHIKV have been elucidated, integration of these cells into 3D scaffolds may prove useful for more fully delineating the effects of CHIKV on each particular cell type. The development of accurate 3D models will allow for the testing of CHIKV effects on human cell lines of individual organ systems, and may be used to research particular symptoms found within those systems. An immunocompetent three-layer model comprised of keratinocytes, fibroblasts, and a central matrix of immune cells in an agarose–fibronectin gel was constructed so that each cell type could be analyzed [92]. This type of fibroblast construct could have potential use as a CHIKV infection model. Similar ideas were demonstrated with another 3D skin model used to demonstrate the effects of fibroblasts against Candida albicans invasion [93].

In light of its association with neonatal encephalitis, 3D fetal and infant brain organoids are of particular note. Human pluripotent stem cell-derived neural organoids are excellent models for fetal brains [94]. These organoids have previously been used to demonstrate the impact of Zika virus (ZIKV) on fetal brain development, with particular emphasis on microcephaly. Human embryonic stem cell-derived cerebral organoids were used to identify a connection between ZIKV Toll-like receptor-3 activation and microcephaly [95]. 3D cerebral organoids have demonstrated the ability to form critical components of the human forebrain, including cortical neurons with markers for all six layers of the human cortex, and have been used to model the effects of ZIKV during various stages of neurogenesis [96]. Using 3D cerebral organoids to study the effects of CHIKV on fetal and infant neural cells may determine through what receptors and pathways CHIKV impacts neural cells and why it exhibits an increased burden on children.

The development of bone and cartilage models could prove a fruitful source for investigating CHIKV, especially the effects of chronic CHIKV, by providing researchers with accurate human joint proliferation, and ischemic bone marrow necrosis stemming from CHIKV [97]. It has been demonstrated that using a mixture of silk fibroin and chitosan creates bone scaffolds that are suited for osteoblast growth [98]. As CHIKV has been used as a model for other alphaviruses, effective use of a 3D model may have larger consequences for studying alphavirus proliferation and invasion.

Nevertheless, due to the modernity of the field, there is currently a dearth of research using 3D models to study neglected tropical diseases, including CHIKV. Many bone and cartilage models currently under investigation are for implantation rather than infection, though there is overlap in the needs of both simulations. In addition, problems such as lack of vascularization and other related issues must be solved before these scaffolds will be able to fully imitate the bone microenvironment.

## 7. Conclusions

**Figure 1:**
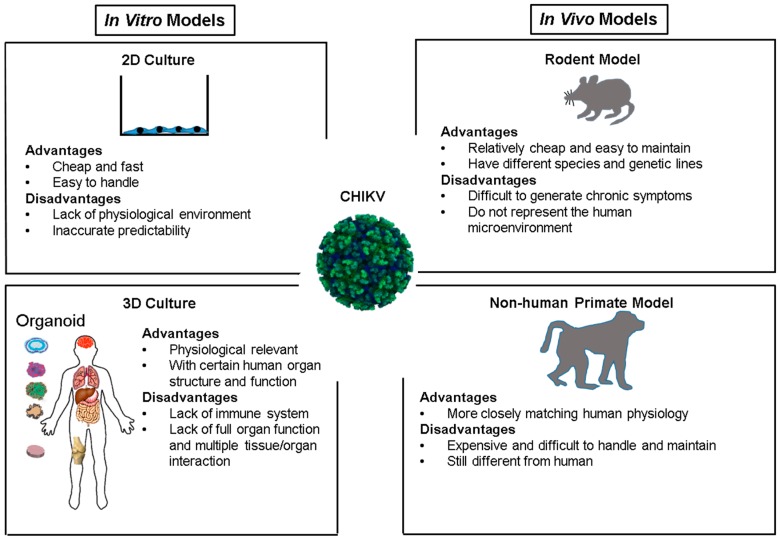
CHIKV in vitro and in vivo models. A comparison of the available infection models to study CHIKV pathogenesis, including advantages and disadvantages for each system. Of note, the 3D culture system has not yet been used as an infection model to study CHIKV infection.

Since the turn of the 21st century, CHIKV has re-emerged as a significant viral threat. The virus has caused outbreaks throughout the world and has demonstrated an ability to spread locally through areas to which it is not endemic. As global travel increases and rates of migration surge, the chance of a larger-scale epidemic will only rise. Though the disease has a low mortality rate, the symptoms and chronic nature of the disease indicate that it should not be ignored. As the disease has not been of great significance for several decades, much knowledge of it remains woefully incomplete. Though more is known now about how the virus enters cells and which cells are susceptible to attack, what occurs once the cells are infected is less known, especially long-term. The development of new models, both animal and synthetic, will aid in more fully realizing treatments for this disease (Figure 1).

In the past decade, new models have been developed to further study CHIKV. Murine and non-human primate models continue to advance and have given a more complete view than ever about the pathogenesis, cellular mechanisms, and replication of the virus. However, though there have been significant advancements in the field of CHIKV animal modeling, all models have significant limitations, and no animal model gives a completely accurate view of the human microenvironment. To that end, 3D scaffolds and tissue development, especially of bone and cartilage cells, may show the most accurate picture of CHIKV infection. As this field is only just beginning to be developed, there is an abundance of room for expansion.

## Figures and Tables

**Table 1:** Recent selected large-scale CHIKV epidemics in the 2000s.

Location	Year/Duration	Affected
Lamu Island, Kenya	2004	13,500
La Réunion	2005–2006	255,000
India	2005	1,380,000
Mauritius	2006	13,500
Gabon	2007	20,000
Thailand	2008–2009	49,000
Republic of Congo	2011	8000
Martinique-Guadeloupe	2014	308,000
French Polynesia	2014–2015	66,000
